# Efficient removal of Eriochrome black-T from aqueous solution using NiFe_2_O_4_ magnetic nanoparticles

**DOI:** 10.1186/s40201-014-0112-8

**Published:** 2014-08-27

**Authors:** Farid Moeinpour, Asma Alimoradi, Maryam Kazemi

**Affiliations:** Department of Chemistry, College of Science, Bandar Abbas Branch, Islamic Azad University, Bandar Abbas, Iran; Department of Chemistry, College of Science, Kerman Branch, Islamic Azad University, Kerman, Iran

**Keywords:** Decolorization, Adsorption, Eriochrome black-T, NiFe_2_O_4_

## Abstract

The magnetic NiFe_2_O_4_ nanoparticles have been synthesized and used as adsorbents for removal of an azo dye, Eriochrome black-T (EBT) from aqueous solution. The NiFe_2_O_4_ nanoparticles were characterized by scanning electron microscope (SEM), Transmission electron microscope (TEM), X-ray diffraction (XRD) and Fourier transform infrared spectra (FTIR). The adsorption studies were carried out under various parameters, such as pH, adsorbent dosage, contact time and initial dye concentration. The experimental results show that the percentage of adsorption increases with an increase in the adsorbent dosage. The maximum adsorption occurred at the pH value of 6.0. The equilibrium uptake was increased with an increase in the initial dye concentration in solution. Adsorption kinetic data were properly fitted with the pseudo-second-order kinetic model. The experimental isotherms data were analyzed using Langmuir and Freundlich isotherm equations. The best fit was obtained by the Langmuir model with high correlation coefficients (R^2^ = 0.9733) with a maximum monolayer adsorption capacity of 47.0 m g/g.

## Introduction

Organic dyes are widely used in various fields and seriously induce water pollution. Most of the industrial dyes are toxic, carcinogenic, and teratogen [1]. Moreover, they are very stable to light, temperature and microbial attack, making them recalcitrant compounds. From an environmental point of view, the removal of synthetic dyes is of great concern. Among several chemical and physical methods, adsorption process is one of the effective techniques that have been successfully employed for color removal from wastewater [2]. In recent years, magnetic nanoparticles have attracted much attention because of their unique magnetic properties and widespread application in different fields such as mineral separation magnetic storage devices, catalysis, magnetic refrigeration system, heat transfer application in drug delivery system, magnetic resonance imaging (MRI), cancer therapy, and magnetic cell separation [3–7]. Magnetic separation has been gradually regarded as a rapid and effective technique for separating magnetic particles [8,9]. It has been used for many applications in biochemistry, cell biology, analytical chemistry, mining, and environmental technology [10]. The advantages of this separation technology are that the harmful ingredients together with the magnetic particles can be eliminated from the polluted system by a simple magnetic field. The application of magnetic nanoparticles in waste water treatment is becoming an interesting area of research. Nanoparticle exhibit good adsorption efficiency especially due to higher surface area and greater active sites for interaction with metallic species and dyes and can easily be synthesized; several researches have used it as an adsorbent [11–19]. Among the various magnetic nanoparticles, most of them inevitably have the drawback of small adsorption capacity, and especially inefficient regeneration of the adsorbents, which limits their practical application. Ni ferrites with general formula (AB_2_O_4_) are one of the most versatile magnetic materials as they have high saturation magnetization, high Curie temperature, chemical stability and relatively high permeability [20]. In the present paper, NiFe_2_O_4_ magnetic nanoparticles have been used for removal of Eriochrome black-T (EBT) which is used for dyeing silk, wool, nylon multifibers after pretreatment with chromium salts. Pure EBT is also used as an indicator in complexometric titrations for determination of Ca^2+^, Mg^2+^ and Zn^2+^ ions and for biological staining. This dye is hazardous as such and its degradation products like Naphtaquinone are still more carcinogenic. A literature survey showed that only few papers have raised the removal of EBT [2,21–26]. Therefore we became interested to develop NiFe_2_O_4_ as an efficient and low-cost adsorbent for removing this azo dye from aqueous solution.

## Experimental

### Materials and methods

All chemicals were obtained commercially and were used as received. Double distilled water was used throughout. X-ray diffraction analysis was carried out using a XRD model Siemens D-5000 diffractometer with Cu Kα radiation (λ = 1.5406 A°) at room temperature. Surface morphology and particle size were studied by scanning electron microscopy (SEM) using a Hitachi S-4800 SEM instrument. Transmission electron microscope (TEM) observation was performed using Hitachi H-7650 microscope at 80 KV. FT-IR spectra were determined as KBr pellets on a Bruker model 470 spectrophotometer.

### Preparation of the NiFe_2_O_4_

The solution of metallic salts FeCl_3_ (160 mL, 1 M) and NiCl_2_ (40 mL, 1 M) was poured as quickly as possible into the boiling alkaline solution [NaOH (1000 mL, 1 M)] under vigorous stirring. Then the solution was cooled and continuously stirred for 90 min. The resulting precipitate was then purified by a four times repeated centrifugation (4000–6000 rpm, 20 min) and decantation.

### Adsorption experiments

The experiments were carried out in conical flasks at room temperature. 40 mL of organic dye solution of known initial concentration was shaked with different mass magnetic composites and different reaction conditions on a shaker at 250 rpm. The initial pH value of the dye solutions were adjusted with 0.1 mol L^−1^ HNO_3_ or 0.1 mol L^−1^ NaOH solution using a pH meter. After magnetic separation using an external magnet, the equilibrium dye concentrations were determined from UV–vis absorbance characteristic with the calibration curve method at the maximum of absorbance (double beam UV/vis spectrophotometer, Shimadzu, Tokyo, Japan; Model 1601; Table 1).All experiments were performed at room temperature (25 ± 1°C). The studied ranges of the experimental variables were as follows: dye concentration (10, 20, 30, 40, 50, 60, 70 mg/L), initial pH of solution (2, 4, 6, 8, 10), adsorbent dosage (0.01, 0.02, 0.05, 0.1, 0.2 g) and contact time (5, 10, 15, 20, 30 min).

**Table 1:** **Physicochemical characteristics of used dye**

**Name**	**Molecular structure**	**M**_**w**_ **(g/mol)**	**λ**_**max**_ **(nm)**
Eriochrome black-T		461.38	489.95

## Results and discussion

### Characterization of NiFe_2_O_4_ magnetic nanoparticles

**Figure 1:**
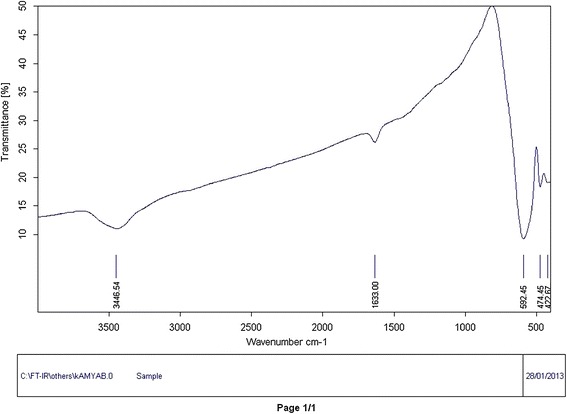
**The FT-IR spectrum of NiFe**_**2**_ **O**_**4**_ **nanoparticles.**

**Figure 2:**
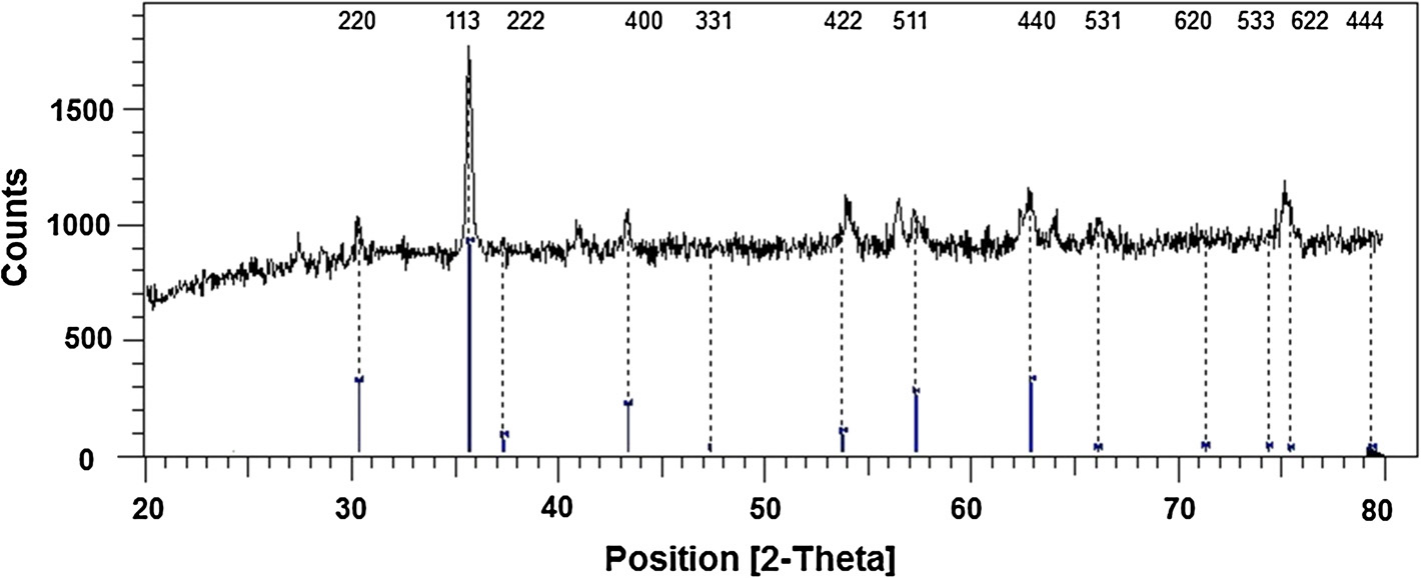
**XRD pattern of NiFe**_**2**_ **O**_**4**_ **nanoparticles.**

**Figure 3:**
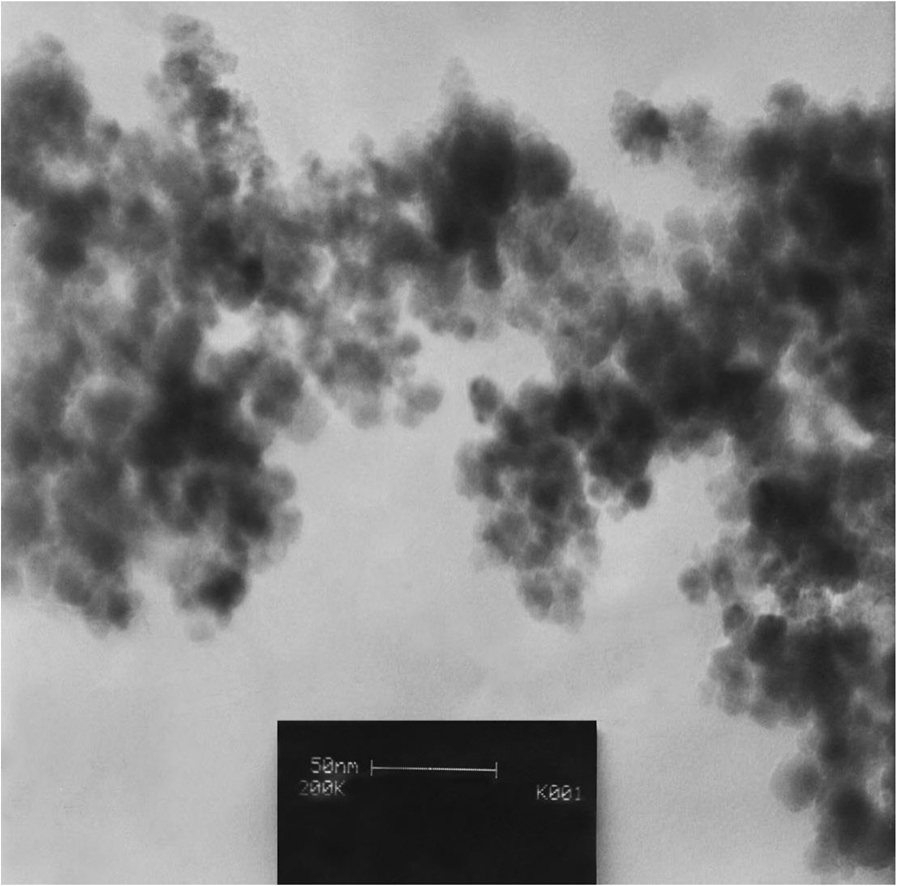
**TEM image of NiFe2O4 nanoparticles.**

**Figure 4:**
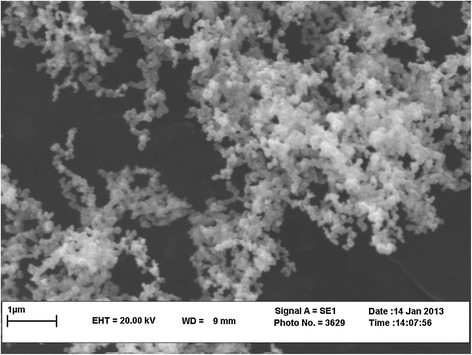
**SEM image of NiFe**_**2**_ **O**_**4**_ **nanoparticles.**

NiFe_2_O_4_ nanocrystallites were prepared according to the reported procedure by R. Massart with slight modifications: fine particles are precipitated in an alkaline solution [27]. NiFe_2_O_4_ nanocrystallites were characterized by FT-IR (Figure 1), XRD (Figure 2) TEM (Figure 3) and SEM (Figure 4). The FT-IR spectrum of NiFe_2_O_4_ (Figure 1) exhibits strong bands in the low-frequency region (1000–500 cm^−1^ ) due to iron oxide skeleton, which is in agreement with the magnetite spectrum. The peak at 1633 cm^−1^ showed the existence of Fe – O and the peak at 3446 cm^−1^ implied the existence of residual hydroxyl groups [28–30]. To confirm the Ni ferrite formation in the synthesized magnetic nanoparticles, the XRD spectrum of the sample was studied. The XRD pattern of the (Figure 2) indicates that these nanoparticles have spinel structure, with all major peaks matching the standard pattern of bulk NiFe_2_O_4_ (JCPDS 10–325). The TEM and SEM photographs of the sample are illustrated in Figure 3 and Figure 4 respectively. Both the SEM and TEM images demonstrate that the prepared magnetic nanoparticles are spherical, narrowly distributed, and well dispersed, with average size of less than 50 nm in the diameter.

### Adsorption and removal of dye from aqueous solution

**Figure 5:**
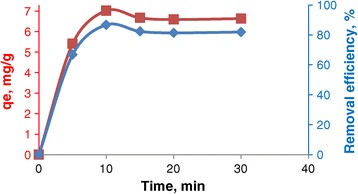
**Effect of contact time.**

The effect of contact time on the amount of EBT adsorbed was investigated at 10 mg L^−1^ initial concentration of dye. It can be observed from Figure 5 that with the increase of contact time, the percentage adsorptions also increased. Minimum adsorption was 66.8% for time 5 minutes to maximum adsorption value 86.7% for the time 10 minutes. The adsorption characteristic indicated a rapid uptake of the dye. The adsorption rate however decreased to a constant value with increase in contact time because of all available sites was covered and no active site available for binding.

The dye removal percentage was calculated as follows [1]:

1 $$ \%\mathrm{removal}=\frac{C_0-{C}_t}{C_0} \times 100 $$

where C_0_ and C_t_ (mg L^−1^) are the concentration of dye in the solution at initial and equilibrium time, respectively.

**Figure 6:**
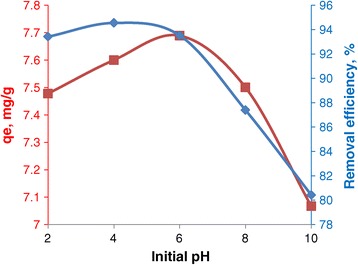
**Percentage of dye removal at different pH.**

The effect of pH on adsorption of dyes was investigated in Figure 6 from pH 2 to 10. Solution pH may affect both aqueous chemistry and surface binding sites of the adsorbent [31]. Removal of EBT increases with increasing pH and a maximum value was found at pH 6.0. The observed trend can be explained by effect of the surface charge of adsorbent. The hydroxyl groups on NiFe_2_O_4_ as revealed by FT-IR spectrum (Figure 1, peak at 3446.54 cm^−1^) play a dominant role in the EBT adsorption. Depending on the solution pH, the adsorbent surface undergoes protonation or deprotonation [32] as shown by the Equation (2):

2 $$ \hbox{--} \mathrm{Ni}\hbox{--}\ \mathrm{Fe}\hbox{--} \mathrm{O}\mathrm{H} + {\mathrm{H}}^{+}\rightleftarrows \hbox{--} \mathrm{Ni}\hbox{--} \mathrm{Fe}\hbox{--} \mathrm{O}\hbox{--} {{\mathrm{H}}_2}^{+} $$

At pH < 7, −Ni–Fe–O–H_2_^+^ is the dominant species. These species having high positive charge density make the negative charged dye (EBT) adsorption favorable due to electrostatic attraction. However, at pH > 7, −Ni– Fe–OH is the dominant species in NiFe_2_O_4_. Such deprotonated species undergo electrostatic repulsion for negative charged dye. This causes decreased dye adsorption [17].

**Figure 7:**
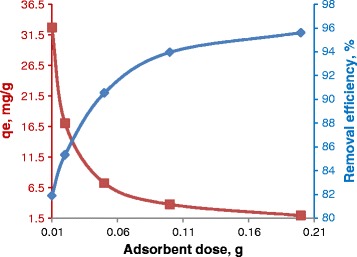
**Effect of adsorbent dosage on adsorption of EBT.**

The effect of variation in the adsorbent amount on the process adsorption of EBT was studied, with different adsorbent amount in the range of 0.01-0.2 g. The results obtained are shown in Figure 7. From Figure 7, it is observed that as the adsorbent dose increases, the percentage removal also increase, until it reaches a saturation point, where the increase in adsorbent dose does not change the percentage removal. An increase in adsorption rate with adsorbent dosage can be attributed to increased surface area and the availability of more adsorption sites [17]. The best removal of EBT is at about 91 %, using an adsorbent dosage of 0.05 g in 10 mg L^−1^ EBT solution.

**Figure 8:**
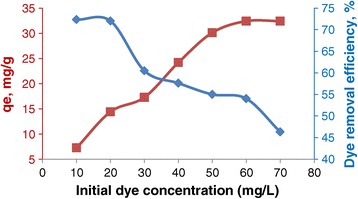
**The effect of different dye equilibrium concentrations to NiFe**_**2**_ **O**_**4**_ **magnetic nanoparticles.**

The maximum adsorption capacities at 298 K in the concentration range studied are 32.41 mg g ^−1^ for EBT (Figure 8). The amount of dye adsorbed (Q_e_) was calculated using the Equation (3) [33]:

3 $$ {\mathrm{Q}}_{\mathrm{e}}=\frac{\left({C}_0-{C}_e\right)\mathrm{V}}{\mathrm{m}} $$

where C_0_ and C_e_ are the initial and equilibrium concentrations of dye (mg L^−1^), m is the mass of NiFe_2_O_4_ nanoparticles (g), and V is the volume of solution (L).

It is obvious from the data that adsorption capacity of EBT dye increases (Figure 8), but the percent removal of EBT decreases with the increase in initial concentration, suggesting that the adsorption of EBT on to NiFe_2_O_4_ is highly dependent on initial dye concentration. Because, the total number of available adsorption sites is fixed for a given adsorbent dose.

#### Adsorption isotherms

Isotherms study can describe how an adsorbate interacts with adsorbent. The experimental data were correlated by Langmuir and Freundlich models. The related linear equations are shown in Equations (4) and (5) respectively:

4 $$ \frac{1}{q_e} = \frac{1}{k_{l\ }{q}_m}\frac{1}{C_e} + \frac{1}{q_m} $$

where q_e_ = the amount of dye adsorbed per unit mass at equilibrium (mg/g); q_m_ = the maximum amount of adsorbent that can be adsorbed per unit mass adsorbent (mg/g); C_e_ = concentration of adsorbent (in the solution at equilibrium (mg/l); k_a_ = adsorption equilibrium constant.

A plot of $\frac{1}{q_e}$ versus $\frac{1}{C_e}$ gives a straight line, with a slope of $\frac{1}{k_{l\ }{q}_m}$ and intercept $\frac{1}{q_m}$.

Freundlich equation:

5 $$ log{q}_e= log{K}_F + \frac{1}{n}\  log{C}_e $$

where C_e_ (mg/ L) and q_e_ (mg/g) are the equilibrium concentration of adsorbent in the solution and the amount of adsorbent adsorbed at equilibrium respectively; K_F_ (mg^1-(1/n)^ L^1/n^ g^−1^) and *n* are the Freundlich constant which show the adsorption capacity for the adsorbent and adsorption intensity, respectively.

A plot of *log q*_*e*_ versus log C_e_ gives a straight line of slope *1/n* and intercept *log K*_F_.

If $\frac{1}{n}<1,$ then the adsorption is favorable. If $\frac{1}{n}>1$ the adsorption bond becomes weak and unfavorable adsorption occurs.

At first, we correlated the adsorption data at different initial concentrations of EBT in terms of the Langmuir isotherm (Equation (4)). Furthermore, we examined the data according to the Freundlich isotherm (Equation (5)). The calculated parameters of the Langmuir and Freundlich models are given in Table 2. The comparison of correlation coefficients (R^2^) of the linearized form of both equations indicates that the Langmuir model yields a better fit for the experimental equilibrium adsorption data than the Freundlich model. This suggests the monolayer coverage of the surface of NiFe_2_O_4_ nanoparticles by EBT molecules.

**Table 2:** **Isotherm and kinetic model parameters for the EBT adsorption on NiFe**_**2**_ **O**_**4**_ **magnetic nanoparticles**

**Isotherms models**						
Langmuir			Freundlich			
R^2^	q_m_ (mg/g)	k_a_ (L/mg)	R^2^	K_F_ (mg^1-(1/n)^ L^1/n^ g^−1^)	1/n	
0.9733	47.0	0.067	0.9382	4.450	0.5913	
Kinetic models						
Pseudo first-order			Pseudo second-order			
R^2^	q_e,cal_ (mg/g)	k_1_ (min^−1^)	R^2^	q_e,_exp. (mg/g)	q_e,_cal. (mg/g)	k_2_ (g/(mg.min))
0.733	3.379	0.093	0.996	7.022	6.971	0.291

The maximum adsorption capacity is compared in Table 3 with the data reported by other authors for EBT adsorption.

**Table 3:** **Maximum adsorption capacities of EBT from aqueous media using various adsorbents**

**Adsorbents**	**q**_**m**_ **(mg/g)**	**References**
NiFe_2_O_4_ nanoparticles	47	This study
Scolymus hispanicusL.	167.77	[2]
Eucalyptus bark	52.37	[21]
Activated carbon prepared from waste rice hulls	160.36	[25]
β-Cyclodextrins/Polyurethane Foam Material	20.17	[26]

#### Adsorption kinetics

In this study, pseudo-first-order and pseudo-second-order kinetics model were applied to examine the controlling mechanism of EBT adsorption from aqueous solutions. Adsorption equilibrium was reached in 10 min (Figure 5). The linear form of the pseudo first-order model and pseudo second-order kinetics model can be described as shown in Equations (6) and (7) respectively:

6 $$ ln\left({q}_e-{q}_t\right)= ln{q}_e-{k}_1.t $$

7 $$ \frac{t}{q_t} = \frac{1}{k_2{q}_e^2} + \frac{t}{q_e} $$

where, q_e_ and q_t_ are the adsorption capacities at equilibrium and at time t (min) respectively. k_1_ (min^−1^) and k_2_ (g/(mg.min)) are the pseudo first-order and pseudo second-order rate constants respectively [34]. The equilibrium experimental results were not well fitted with the pseudo first-order model (Table 2). The values of q_e_ and k_2_ can be calculated from the slope and intercept of the plot of t/q_t_ versus t (figure was not shown). The results listed in Table 1 show that the correlation coefficient is very high (R^2^ = 0.9962). Furthermore, the calculated equilibrium adsorption capacity was consistent with the experimental result. This result suggested that the kinetics data were better described with a pseudo second-order kinetics model.

## Conclusion

NiFe_2_O_4_ magnetic nanoparticles with average size less than 50 nm in the diameter have been synthesized for removal of an azo dye from water. The prepared magnetic nanoparticles can be well dispersed in the aqueous solution and easily separated from the solution using an external magnet after adsorption. The adsorption capacity for EBT in the concentration range studied is 32.41 mg g^−1^. The process of purifying water pollution presented here is clean and safe using the magnetic nanoparticles. Therefore, this adsorbent was found to be useful and valuable for controlling water pollution due to dyes.
